# Awareness and early implementation of the European AIDS Clinical Society Guidance on statin use in HIV for the primary prevention of cardiovascular disease: A cross‐sectional survey of HIV clinicians in Europe

**DOI:** 10.1111/hiv.70271

**Published:** 2026-06-18

**Authors:** Jasmini Alagaratnam, Abiu Sempere, Susanne Dam Nielsen, Esteban Martinez

**Affiliations:** ^1^ Department of HIV Medicine & Sexual Health Chelsea & Westminster Hospital NHS Foundation Trust London UK; ^2^ Section of Virology, Department of Infectious Disease Imperial College London London UK; ^3^ HIV Unit Infectious Diseases Service, Hospital, Clinic‐Fundacio de recerca Clinic Barcelona – Institut de investigacions Biomediques August Pi i Sunyer Barcelona Spain; ^4^ Department of Infectious Diseases Copenhagen University Hospital Rigshospitalet Denmark; ^5^ CIBERINFEC, Instituto de Salud Carlos III Madrid Spain; ^6^ Reial Acadèmia de Medicina de Catalunya Barcelona Spain

**Keywords:** HIV, implementation, prevention cardiovascular disease, statins, survey

## Abstract

**Objectives:**

Cardiovascular disease (CVD) is a major comorbidity and leading cause of morbidity and mortality in people with HIV (PWH). The REPRIEVE trial demonstrated that statin therapy significantly reduces cardiovascular events in PWH, prompting updated recommendations from major international societies. We assessed awareness and early implementation of the European AIDS Clinical Society (EACS) statin guidance among HIV clinicians in Europe.

**Materials and Methods:**

We conducted an anonymous cross‐sectional online survey among members of the EACS Young Investigators Network Group (YING). The questionnaire evaluated clinician characteristics, reported awareness and implementation of statin guidance, cardiovascular risk assessment practices, prescribing patterns and perceived barriers. Descriptive analyses were performed.

**Results:**

Of 125 individuals invited to take part in the survey, 22 (18%) physicians from multiple European countries responded. All respondents were aware of the EACS statin guidance, and 91% reported that it influenced clinical practice. Cardiovascular risk was routinely assessed by most clinicians (77% always or often), with HIV physicians primarily responsible for statin prescribing (59%). Structural barriers reported included reimbursement limitations, fragmented care pathways and patient‐level barriers, such as low perceived CVD risk and statin hesitancy.

**Conclusions:**

Awareness of EACS statin guidance among this cohort of HIV clinicians in Europe who are YING members is reported as high, but implementation gaps remain. Addressing structural and patient‐level barriers and improving guideline implementation will be essential to optimize CVD prevention in PWH.

## INTRODUCTION

Cardiovascular disease (CVD) has emerged as a major comorbidity in people with HIV, and is now a leading contributor to morbidity and mortality in this population, with consistently higher incidence compared with the general population despite effective antiretroviral therapy [1, 2, 3]. Multiple mechanisms contribute to this increased risk, including chronic inflammation, immune activation, metabolic abnormalities, and higher prevalence of traditional cardiovascular risk factors [4, 5].

Traditional cardiovascular risk prediction models generally underestimate cardiovascular risk among people with HIV because they do not incorporate HIV‐specific risk factors, potentially contributing to undertreatment with preventive interventions such as statins [5, 6].

The Randomized Trial to Prevent Vascular Events in HIV (REPRIEVE) trial demonstrated that pitavastatin significantly reduced major adverse cardiovascular events in people with HIV receiving antiretroviral therapy, including individuals at low‐to‐moderate estimated cardiovascular risk [7]. These findings represent a major advance in cardiovascular prevention in HIV.

Following REPRIEVE, the European AIDS Clinical Society (EACS) issued interim and subsequently comprehensive guidance recommending statin therapy for primary prevention of CVD in people with HIV [8]. Similar recommendations were issued by the US Department of Health and Human Services (DHHS) [9, 10], and national societies, including the British HIV Association (BHIVA) and Grupo de Estudio del SIDA (GeSIDA) (Spain) [11, 12].

However, despite the publication of updated recommendations, there are currently limited data regarding clinician awareness, perceptions and early implementation of statin guidance in routine HIV clinical practice across Europe. Understanding these factors is important to identify potential barriers to implementation and inform strategies to improve cardiovascular prevention in people with HIV. We therefore conducted a cross‐sectional survey among HIV clinicians working in Europe to assess reported awareness, perceptions and early implementation of statin therapy for the primary prevention of CVD following publication of the REPRIEVE trial [7] and subsequent guidance from EACS [8].

## MATERIALS AND METHODS

### Study design and population

We conducted an anonymous exploratory cross‐sectional descriptive online survey among members of the Young Investigators Network Group (YING), a Europe‐wide network of early‐career HIV clinicians and researchers actively involved in HIV clinical care, research and scientific collaboration and is affiliated to EACS [13].

The survey was distributed electronically between September and October 2025. Participation was voluntary and anonymous. Ethics approval was not required because the survey collected anonymous data from healthcare professionals.

### Questionnaire design

The questionnaire was purpose‐designed by author EM with input from the other co‐authors to assess clinician awareness and influence of statin guidance, cardiovascular risk assessment and prescribing practices and structural and patient‐level barriers. The questionnaire, conducted in English, consisted predominantly of 9 participant characteristic questions, 23 structured multiple‐choice ‘best answer’ questions, alongside two optional free‐text response questions. Free‐text responses were analysed via thematic clustering, grouping the data into themes. The full questionnaire is provided in Appendix 1.

Prior to dissemination, the questionnaire underwent review and pilot testing among senior members of the EACS YING steering committee to assess clarity, readability and interpretability, with subsequent refinements made based on feedback received. Once the survey window was open, an email invitation containing the survey link was distributed to all members of the EACS Young Investigators (YING) network. No participant sub‐selection was performed.

### Statistical analysis

Descriptive statistics were used. Categorical variables were summarized as frequencies and percentages.

## RESULTS

Of 125 individuals invited to take part in the survey, 22 (18%) physicians from multiple European countries participated, representing diverse healthcare systems and clinical settings. Overall, 4 (18%) respondents were from the United Kingdom, 3 (13%) each from Portugal, Spain and Turkey, and 9 (41%) from Italy, Austria, Denmark, Belgium, Ireland, Netherlands, Serbia and Switzerland. In total, 13 (59%) self‐identified as female, and 9 (41%) as male. Most respondents were aged 30–39 years (15, 68%), and all were actively involved in HIV clinical care.

All survey respondents reported being aware of the EACS statin guidance. Most clinicians reported that the guidance influenced their clinical practice, with 41% reporting a significant impact and 50% reporting some impact, while only a minority reported minimal or no impact on their clinical practice. EACS guidance was perceived as the most influential source of recommendations in most settings, although other national or international guidelines were also used. Cardiovascular risk assessment was reported to be routinely performed by most clinicians, with 18% reporting always assessing risk and 59% often assessing risk. However, variability in risk assessment approaches was observed. Half of clinicians reported using general population risk scores, while fewer reported using HIV‐specific tools or combining both approaches. In addition, primary care involvement in cardiovascular risk management was reported as inconsistent. These findings are summarized in Table 1.

**TABLE 1:** Awareness and influence of cardiovascular prevention guidelines and cardiovascular risk assessment practices.

Variable	*n* (%)
Awareness of cardiovascular prevention among HIV physicians	
Most are aware	15 (68)
Some are aware	6 (27)
Few are aware	1 (4)
Awareness of cardiovascular prevention among people with HIV	
Most are aware	5 (23)
Some are aware	9 (41)
Few are aware	7 (32)
None are aware	1 (4)
Impact of EACS statins guidance on the respondent's clinical practice	
Significant impact	9 (41)
Some impact	11 (50)
Minimal impact	1 (4)
No impact	1 (4)
Influence of EACS statin guidance on overall clinical practice in the respondent's setting	
A great deal	9 (41)
Somewhat	9 (41)
A little	1 (4)
Not at all	2 (9)
Don't know	1 (4)
Guidelines influencing overall clinical practice other than the EACS statin guidance in the respondent's setting	
BHIVA rapid guidance	5 (23)
US DHHS recommendations	1 (4)
GeSIDA (Spain) position paper	1 (4)
European cardiology guidelines	1 (4)
Availability of national or local cardiovascular prevention programmes	
National programmes	4 (18)
Local programmes	9 (41)
Both national and local programmes	1 (4)
None	6 (27)
Not sure	2 (9)
Primary care involvement in cardiovascular risk management	
Routinely	1 (4)
Sometimes	11 (50)
Rarely	9 (41)
Never	1 (4)
Routine cardiovascular risk assessment during HIV consultations	
Always	4 (18)
Often	13 (59)
Sometimes	4 (18)
Rarely	1 (4)
Cardiovascular risk scores used	
General population scores	11 (50)
HIV‐specific scores	8 (36)
Both	3 (14)

Abbreviations: BHIVA, British HIV Association; EACS, European AIDS Clinical Society; GeSIDA, Grupo de Estudio de SIDA; US DHHS, United States Department of Health and Human Services.

HIV physicians were the primary prescribers of statins in most settings (59%), followed by primary care physicians and other specialists. Statin availability and reimbursement varied across settings, with only half of respondents reporting full reimbursement. These structural and healthcare system factors are summarized in Table 2.

**TABLE 2:** Statin prescribing and structural factors, and patient‐related factors affecting statin use, in the respondent's setting.

Variable	*n* (%)
Primary prescriber of statins	
HIV physician	13 (59)
Primary care physician	6 (27)
Cardiologist	2 (9)
Endocrinologist	1 (4)
Statin reimbursement policy	
Fully covered	11 (50)
Partially covered	11 (50)
Statin availability	
All statins available	10 (46)
Limited availability	11 (50)
Not sure	1 (4)
Financial barriers to statin access	
None	6 (27)
Very few barriers	8 (36)
Some barriers	6 (27)
Not sure	2 (9)
Patient concerns regarding statin initiation	
Always	1 (4)
Often	11 (50)
Sometimes	9 (41)
Rarely	1 (4)
Patients declining statin initiation	
Frequently	4 (18)
Occasionally	13 (59)
Rarely	5 (23)
Patient adherence and tolerance to statins	
Good	11 (50)
Moderate	11 (50)
Serious statin toxicity observed	
Frequently	1 (4)
Rarely	11 (50)
Never	9 (41)
Not sure	1 (4)
Respondent's concern regarding statin–ART drug interactions	
Very concerned	2 (9)
Somewhat concerned	4 (18)
Not concerned	16 (73)
Respondent's perceived benefits beyond cardiovascular prevention	
Multiple additional benefits	7 (32)
Some additional benefits	12 (55)
Only cardiovascular prevention	3 (14)
Smoking assessment during HIV consultations	
Always	11 (50)
Often	10 (46)
Sometimes	1 (4)
Smoking cessation interventions offered	
Always	6 (27)
Often	9 (41)
Sometimes	6 (27)
Rarely	1 (4)
Smoking cessation reimbursement	
Fully covered	5 (23)
Partially covered	13 (59)
Not covered	2 (9)
Not sure	2 (9)
Patient uptake of smoking cessation services	
Most patients access services	1 (4)
Some patients access services	9 (41)
Rarely access services	11 (50)
Not sure	1 (4)

Abbreviation: ART, antiretroviral treatment.

Patient‐level barriers were commonly reported. These included concerns regarding statin adverse effects and challenges related to adherence and tolerance. Despite these concerns, statin tolerance was generally reported as good, and serious statin‐related adverse effects were uncommon. These patient‐related factors are summarized in Table 2.

Within the free text section of the survey, challenges to implementing CVD prevention for people with HIV clustered into three main themes: (1) structural barriers, notably medication cost or reimbursement concerns, inequitable access to primary care and prevention services (e.g., smoking cessation), and resource constraints in more deprived settings; (2) clinician and service barriers, such as limited consultation time, knowledge gaps, inconsistent guideline use, and weak coordination between HIV and primary care services; and (3) patient‐level barriers, including low perception of CVD risk, statin hesitancy driven by fear of side effects, adherence difficulties and pill burden. Strategies proposed by the respondents to improve CVD prevention and management consistently emphasized education and awareness through accessible resources, clearer communication of evidence and support for healthy lifestyle modifications. The need for stronger service integration was also highlighted, including closer collaboration between HIV specialists, primary care and cardiology, alongside the use of CVD risk prediction tools and multidisciplinary care. Finally, system‐level supports such as improved reimbursement, wider statin access and expanded prevention services (e.g., smoking cessation, digital tools) were seen as key to improving CVD risk management among people with HIV.

## DISCUSSION

This study provides insight into early awareness and the implementation of EACS statin guidance among European HIV clinicians who are YING members, following publication of the REPRIEVE trial [7]. Reported awareness among survey respondents was high, likely reflecting rapid dissemination of REPRIEVE findings and subsequent guideline updates across European HIV clinical networks [8, 11, 12]. However, despite high reported awareness, challenges in implementation remain.

Risk assessment practices were heterogeneous, and HIV‐specific cardiovascular risk tools were inconsistently used. This is clinically relevant because traditional risk prediction models underestimate cardiovascular risk in people with HIV and may contribute to undertreatment [5, 6]. Greater integration of cardiovascular risk assessment into routine HIV care may therefore be necessary to ensure appropriate identification of patients who could benefit from statin therapy.

Participants frequently reported structural barriers, including reimbursement limitations and fragmented care coordination, and highlighted the importance of accessible educational resources and clearer communication of evidence to support implementation in routine practice. These survey findings may help explain the implementation gaps observed in real‐world studies following publication of the REPRIEVE trial. For example, a real‐world study presented at the EACS Conference 2025 showed that statin prescribing increased following REPRIEVE but remained suboptimal in eligible individuals [14]. Similarly, a clinical audit demonstrated that statins were initiated in only a minority of eligible patients despite updated recommendations [15] and that when statins were prescribed, low‐density lipoprotein (LDL) target achievements remained suboptimal [16, 17]. While these studies did not specifically evaluate the underlying causes of incomplete implementation, our findings suggest that clinician‐reported structural barriers, including reimbursement limitations and fragmented care pathways, may contribute to these observed gaps in routine clinical care. Taken together, these findings suggest that although statin prescribing has increased following publication of the REPRIEVE trial and updated recommendations, implementation remains suboptimal in routine clinical care.

Patient‐level barriers were also frequently reported, including low perceived cardiovascular risk and concerns about adverse effects. These findings are consistent with previous studies demonstrating that patient perception and adherence are key determinants of preventive therapy implementation [18, 19]. Improving patient education and clinician–patient communication may therefore represent an important component of successful implementation.

These findings should be interpreted in the context of evolving clinical practice following REPRIEVE. Multiple international guidelines, including EACS, DHHS, BHIVA and GeSIDA (Spain), now recommend broader use of statins for primary prevention in people with HIV [8, 9, 10, 11, 12]. However, real‐world implementation remains incomplete, highlighting the need for targeted implementation strategies. Dissemination of guideline recommendations alone is insufficient, and implementation efforts must address structural, clinician and patient‐level barriers. Integration of cardiovascular prevention into routine HIV care pathways may help reduce implementation gaps.

Strengths of this study include the participation of clinicians from multiple European countries and a focus on physicians actively involved in HIV care, providing insight into early implementation in clinical practice. Limitations include the small sample size and the potential for selection bias. The survey was distributed among YING members, who mainly comprise early‐career‐stage HIV clinicians, who may be more up to date with emerging evidence and new guidelines, including findings from the REPRIEVE trial [7]. This likely results in a cohort with higher awareness and/or earlier adoption of updated statin‐based cardiovascular primary prevention strategies than among the wider HIV clinician community, and therefore, awareness may be overestimated. Our findings should therefore be interpreted as exploratory and may not be fully representative of all clinicians providing HIV care across Europe.

In contrast, reported implementation practices may reflect more broadly applicable structural and system‐level challenges, although this cannot be definitively surmised from the present study's findings. Notably, even within this population of highly engaged HIV clinicians, implementation of the EACS guidance on statins use was suboptimal, highlighting the persistent gap between emerging evidence and implementation into routine clinical practice. Survey participants also reported knowledge gaps and the need for improved education and accessible resources as key barriers, which further supports concerns that awareness may be even lower in less specialized or less research‐engaged clinical populations.

Several survey items captured clinicians' perceptions of awareness and practice among other stakeholders, rather than directly measuring behaviours or knowledge in those groups. These included questions relating to awareness of CVD prevention among people living with HIV and other healthcare professionals, the influence of EACS guidance on national or local practice, and the extent to which cardiovascular risk is addressed by different providers. As such, these responses reflect perceived rather than objectively measured levels of awareness or practice, introducing some uncertainty in interpretation.

Patient and public involvement (PPI) was not incorporated into the design of the survey. Future studies would benefit from involving PPI to further enhance the relevance and comprehensiveness of questionnaire development. Future work is planned to expand the survey to a broader group of HIV clinicians across different settings, including direct assessment of all relevant stakeholder groups to more comprehensively evaluate awareness, attitudes and implementation of CVD prevention strategies.

In conclusion, awareness of EACS statin guidance was reportedly high among YING members who responded to the survey, although respondents continued to report structural and practical barriers that may affect consistent implementation in routine clinical care. This gap between evidence and implementation is a common challenge in preventive medicine and highlights the need for targeted strategies beyond the publication of guideline recommendations. As CVD continues to represent a major contributor to morbidity and mortality in people with HIV, effective implementation of preventive strategies will become increasingly important in routine HIV care. Efforts to improve cardiovascular prevention in people with HIV should focus on facilitating guideline uptake, addressing structural barriers, and facilitating broader dissemination of cardiovascular prevention strategies across HIV care settings.

## AUTHOR CONTRIBUTIONS

*Conceptualization*: EM. *Design of the questionnaire*: EM, SDN and JA. *Data curation and validation*: JA. *Contribution to the design of the data analysis*: EM, JA and SDN. *Data analysis*: JA. *Supervision*: EM and SDN. *Writing of the first draft of the manuscript*: JA. All authors contributed to the review and editing of the final submitted manuscript for intellectual content.

## CONFLICT OF INTEREST STATEMENT

SDN has received grants from the Novo Nordisk foundation, Independent Research Fund Denmark, and Svend Andersen Fonden; personal fees from Gilead; and has served on advisory boards for GSK, Gilead and Takeda. The remaining authors declare no conflicts of interest related to this work.

## Data Availability

The data that support the findings of this study are available on request from the corresponding author. The data are not publicly available due to privacy or ethical restrictions.

## APPENDIX 1 SURVEY QUESTIONNAIRE

### 1.1 EACS survey—Implementation and perceptions of primary prevention of cardiovascular disease in people with HIV (statins)

#### 1.1.1 Structured list of survey items, response formats and answer options

**Table:**

**Questionnaire structure**: The survey included demographic/background questions followed by nine thematic sections. Most items used single‐answer multiple‐choice formats; frequency questions used ordered response options; the final section used open free‐text responses. The country question was presented as a country field/dropdown list in the online form.

##### 1.1.1.1 Demographics/background

1. What is your gender?
  **Response format**: Multiple choice
  **Response options**:
  - Female
  - Male
  - Non‐binary
  - Prefer not to say
  - Other
2. What is your current healthcare role?
  **Response format**: Multiple choice
  **Response options**:
  - Physician
  - Nurse
  - Pharmacist
  - Other
3. In which country do you currently live?
  **Response format:** Country field/dropdown list
  **Note**: The online form used a country list.
4. Please select your age group:
  **Response format**: Multiple choice
  **Response options**:
  - <30 years
  - 30–39 years
  - 40–49 years
  - 50–59 years
  - 60–69 years
  - ≥70 years
  - Prefer not to say
5. What type of healthcare setting do you mainly work in? Please select the option that best reflects your primary role.
  **Response format**: Multiple choice
  **Response options**:
  - HIV clinic
  - Primary care
  - Other
6. What type of healthcare facility do you primarily work in? Please select the option that best matches your main role.
  **Response format**: Multiple choice
  **Response options**:
  - Public hospital
  - Public outpatient clinic/health centre
  - Private practice/clinic
  - Other
7. How many years have you been providing healthcare to people living with HIV?
  **Response format**: Multiple choice
  **Response options**:
  - <5 years
  - 5–10 years
  - 11–20 years
  - 21–30 years
  - >30 years
8. Approximately how many people living with HIV have you interacted with in your professional role?
  **Response format**: Multiple choice
  **Response options**:
  - None
  - <25
  - <50
  - 50–199
  - 200–499
  - ≥500
  - Don't know/not applicable
9. Do you take part in clinical decision‐making related to cardiovascular disease prevention (e.g., risk assessment, prescribing medications, lifestyle counselling) for people living with HIV?
  **Response format**: Multiple choice
  **Response options**:
  - Yes, regularly
  - Yes, occasionally
  - Yes, rarely
  - No

##### 1.1.1.2 Section 1: Guideline awareness and local impact

10 Are you familiar with the European AIDS Clinical Society guidance for the primary prevention of cardiovascular disease: Statin use in HIV?
  **Response format**: Multiple choice
  **Note**: The form included the following reference link: https://www.thelancet.com/journals/lanhiv/article/PIIS2352-3018(25)00047-5/fulltext
  **Response options**:
  - Yes
  - No
  - Don't know/not sure
11 To what extent do the EACS guidelines influence national guidelines or clinical practice in your country?
  **Response format**: Multiple choice
  **Response options**:
  - A great deal
  - Somewhat
  - A little
  - Not at all
  - Don't know/not sure
12 Do the EACS guidelines on cardiovascular disease prevention affect the care of people living with HIV in your workplace or setting?
  **Response format**: Multiple choice
  **Response options**:
  - Yes, a significant impact
  - Yes, some impact
  - Minimal impact
  - No impact
  - Not sure/don't know

##### 1.1.1.3 Section 2: Physician and patient awareness of primary prevention of cardiovascular disease

13 In your experience, are HIV physicians in your country aware of cardiovascular disease prevention for people living with HIV?
  **Response format**: Multiple choice
  **Response options**:
  - Yes, most are aware
  - Yes, some are aware
  - No, few are aware
  - No, none are aware
  - Not sure/don't know
14 In your experience, are people living with HIV in your country aware of cardiovascular disease prevention?
  **Response format**: Multiple choice
  **Response options**:
  - Yes, most are aware
  - Yes, some are aware
  - No, few are aware
  - No, none are aware
  - Not sure/don't know

##### 1.1.1.4 Section 3: Prevention programmes and risk assessment

15 Are there any national or local programmes specifically dedicated to the prevention of cardiovascular disease in people living with HIV?
  **Response format**: Multiple choice
  **Response options**:
  - Yes, national programmes
  - Yes, local programmes
  - Yes, both national and local programmes
  - No
  - Not sure/don't know
16 Do you routinely assess cardiovascular disease risk during your clinical consultations with people with HIV?
  **Response format**: Multiple choice/frequency scale
  **Response options**:
  - Never
  - Rarely
  - Sometimes
  - Often
  - Always
17 Which tools or cardiovascular risk scores do you use to assess cardiovascular disease risk in people living with HIV?
  **Response format**: Multiple choice
  **Response options**:
  - None
  - General population scores
  - HIV‐specific scores
  - Both
  - Don't know
18 Do you routinely ask people living with HIV about their smoking habits during consultations?
  **Response format**: Multiple choice/frequency scale
  **Response options**:
  - Never
  - Rarely
  - Sometimes
  - Often
  - Always
19 If a patient smokes, do you offer smoking cessation interventions?
  **Response format**: Multiple choice/frequency scale
  **Response options**:
  - Never
  - Rarely
  - Sometimes
  - Often
  - Always

##### 1.1.1.5 Section 4: Access, coverage and costs

20 Are smoking cessation programmes and medications (e.g., varenicline) covered by health insurance/social security?
  **Response format**: Multiple choice
  **Response options**:
  - Yes, fully
  - Partially
  - No
  - Don't know
21 Are statins for the primary prevention of cardiovascular disease covered by health insurance or social security in your country?
  **Response format**: Multiple choice
  **Response options**:
  - Yes, fully
  - Partially
  - No
  - Don't know
22 Are there financial barriers that prevent patients from accessing cardiovascular prevention interventions or medications?
  **Response format**: Multiple choice
  **Response options**:
  - None
  - Very few barriers
  - Yes, some barriers
  - Yes, significant barriers
  - Don't know

##### 1.1.1.6 Section 5: Role of different providers

23 In your experience, do primary care physicians or other specialists address cardiovascular risk in people living with HIV in your country?
  **Response format**: Multiple choice
  **Response options**:
  - Yes, routinely
  - Yes, sometimes
  - Rarely
  - Never
  - Not sure/don't know
24 In your clinical setting, who usually prescribes statins for people living with HIV?
  **Response format**: Multiple choice
  **Response options**:
  - HIV physician
  - Primary care physician
  - Cardiologist
  - Don't know
  - Other

##### 1.1.1.7 Section 6: Patient perceptions and adherence

25 In your experience, do patients express concerns about starting statins, such as pill burden, cost, or potential side effects?
  **Response format**: Multiple choice/frequency scale
  **Response options**:
  - Never
  - Rarely
  - Sometimes
  - Often
  - Always
26 Do patients decline statins due to personal preference?
  **Response format**: Multiple choice
  **Response options**:
  - Yes, frequently
  - Yes, occasionally
  - Rarely
  - Never
  - Not sure/don't know
27 In your experience, how would you describe adherence to and tolerance of statins among patients already taking them?
  **Response format**: Multiple choice
  **Response options**:
  - Excellent adherence and tolerance
  - Good adherence and tolerance
  - Moderate adherence and tolerance
  - Poor adherence and tolerance
  - Not sure/don't know
28 In your experience, do patients referred or signposted to smoking cessation services actually access these services?
  **Response format**: Multiple choice
  **Response options**:
  - Yes, most patients access the services
  - Yes, some patients access the services
  - Rarely
  - Never
  - Not sure/don't know
29 Have you encountered serious cases of statin‐related toxicity in people with HIV?
  **Response format**: Multiple choice
  **Response options**:
  - Yes, frequently
  - Yes, occasionally
  - Rarely
  - Never
  - Not sure/don't know

##### 1.1.1.8 Section 7: Drug interactions and availability

30 Are you concerned about potential drug–drug interactions between statins and antiretroviral therapy (ART)?
  **Response format**: Multiple choice
  **Response options**:
  - Yes, very concerned
  - Yes, somewhat concerned
  - No, not concerned
  - Not sure/don't know
31 Are all types of statins available in your country?
  **Response format**: Multiple choice
  **Response options**:
  - Yes, all statins are available
  - Only some statins are available
  - No, very limited availability
  - Not sure/don't know

##### 1.1.1.9 Section 8: Broader expectations

32 Do you expect statins to provide benefits beyond primary prevention of cardiovascular disease?
  **Response format**: Multiple choice
  **Response options**:
  - Yes, multiple additional benefits
  - Yes, some additional benefits
  - No, only for cardiovascular prevention
  - Not sure/don't know

##### 1.1.1.10 Section 9: Open perspectives

33 In your experience, what are the main challenges to implementing cardiovascular disease prevention for people living with HIV in your country? Please describe any barriers you have observed.
  **Response format**: Open free‐text response
34 What additional measures do you think could improve the prevention and management of cardiovascular risk in people living with HIV? Please share any suggestions related to clinical practice, policy, education, or patient support that could enhance cardiovascular risk prevention in this population.
  **Response format**: Open free‐text response
